# A Soluble Version of Nipah Virus Glycoprotein G Delivered by Vaccinia Virus MVA Activates Specific CD8 and CD4 T Cells in Mice

**DOI:** 10.3390/v12010026

**Published:** 2019-12-24

**Authors:** Georgia Kalodimou, Svenja Veit, Sylvia Jany, Ulrich Kalinke, Christopher C. Broder, Gerd Sutter, Asisa Volz

**Affiliations:** 1 Institute for Infectious Diseases and Zoonoses, LMU Munich, 80539 Munich, Germany; georgia.kalodimou@micro.vetmed.uni-muenchen.de (G.K.); svenja-veit@web.de (S.V.); sylvia.jany@micro.vetmed.uni-muenchen.de (S.J.); asisa.volz@micro.vetmed.uni-muenchen.de (A.V.); 2 German Center for Infection Research (DZIF), partner site Munich, 80539 Munich, Germany; 3 Institute for Experimental Infection Research, TWINCORE, Centre for Experimental and Clinical Infection Research, a joint venture between the Helmholtz Centre for Infection Research Braunschweig and the Hannover Medical School, 30625 Hannover, Germany; Kalinke.Ulrich@mh-hannover.de; 4 Department of Microbiology and Immunology, Uniformed Services University of the Health Sciences, Bethesda, MD 20814, USA; christopher.broder@usuhs.edu

**Keywords:** emerging viruses, vaccination, MVA vector vaccines, T cell responses

## Abstract

Nipah virus (NiV) is an emerging zoonotic virus that is transmitted by bats to humans and to pigs, causing severe respiratory disease and often fatal encephalitis. Antibodies directed against the NiV-glycoprotein (G) protein are known to play a major role in clearing NiV infection and in providing vaccine-induced protective immunity. More recently, T cells have been also shown to be involved in recovery from NiV infection. So far, relatively little is known about the role of T cell responses and the antigenic targets of NiV-G that are recognized by CD8 T cells. In this study, NiV-G protein served as the target immunogen to activate NiV-specific cellular immune responses. Modified Vaccinia virus Ankara (MVA), a safety-tested strain of vaccinia virus for preclinical and clinical vaccine research, was used for the generation of MVA–NiV-G candidate vaccines expressing different versions of recombinant NiV-G. Overlapping peptides covering the entire NiV-G protein were used to identify major histocompatibility complex class I/II-restricted T cell responses in type I interferon receptor-deficient (IFNAR−/−) mice after vaccination with the MVA–NiV-G candidate vaccines. We have identified an H2-b-restricted nonamer peptide epitope with CD8 T cell antigenicity and a H2-b 15mer with CD4 T cell antigenicity in the NiV-G protein. The identification of this epitope and the availability of the MVA–NiV-G candidate vaccines will help to evaluate NiV-G-specific immune responses and the potential immune correlates of vaccine-mediated protection in the appropriate murine models of NiV-G infection. Of note, a soluble version of NiV-G was advantageous in activating NiV-G-specific cellular immune responses using these peptides.

## 1. Introduction

Nipah virus (NiV) is an emerging zoonotic pathogen of global concern that was ranked recently by the World Health Organization (WHO) as a high-priority pathogen. NiV is a negative-sense, single-stranded RNA virus that is a member of the genus *Henipavirus* (family *Paramyxoviridae*). NiV was first identified during a large outbreak affecting humans and pigs in Malaysia and Singapore in 1999 [1]. From 2001 onwards, seasonal outbreaks are observed almost annually in Bangladesh and sporadically in India [2]. Recent outbreaks occurred in Kerala in May 2018 and June 2019, the first time NiV emerged in southern India [2]. Two strains of NiV have been identified, Malaysia and Bangladesh strains, which share 91.8% sequence homology [3]. NiV causes severe respiratory disease and encephalitis [4,5,6], with average case fatality rates (CFR) of 40–75% [7]. Moreover, long-term neurological sequelae and even relapse of encephalitis are observed in many survivors of infections with both strains of NiV [8,9,10].

The natural reservoir of NiV and Hendra virus (HeV) are the fruit bats of the genus *Pteropus*, which are widely distributed in Asia, Australasia, and parts of Africa [11,12]. Moreover, NiV has a broad species tropism and can cause disease in a many animal species [13,14,15]. NiV can infect humans via several routes, which include the consumption of food contaminated with bat secretions [16], transmission from amplification hosts such as pigs [4], and direct human-to-human transmission between very close contacts [17]. Currently there are no licensed treatments or preventative vaccines available for use in humans, which make the development of effective prophylactic measures imperative.

Evidence to date indicates that the *Henipavirus* glycoprotein G is a highly promising target of virus-neutralizing antibodies to counteract infections with highly pathogenic henipaviruses. The G glycoproteins of NiV and HeV share 83% amino acid sequence homology and are type II transmembrane proteins [18]. The glycoproteins of both viruses bind to the host cell receptors ephrin-B2 and ephrin-B3 [19,20,21,22], which are highly conserved across many species [23]. To date, the most promising therapeutic approach against *Henipavirus* infection is the one based on the application of the experimental human monoclonal antibody m102.4, which binds the ephrin-B2 and ephrin-B3 receptor-binding site on the glycoproteins of HeV (HeV-G) and NiV (NiV-G) [24]. The protective efficacy of m102.4 has been successfully evaluated in different preclinical models including ferrets and African green monkeys (AGM) [25,26,27].

Currently, several vaccines delivering NiV-G have been shown to protect against lethal challenge infections in preclinical testing. These candidate vaccines include recombinant viruses developed using vaccinia virus [28], canarypox [29], vesicular stomatitis virus (VSV) [30,31,32,33,34], rabies virus [35], measles virus [36], and adenovirus platforms [37,38]. To date, the only licensed *Henipavirus* vaccine is the equine vaccine against HeV, Equivac^®^ HeV, which was approved for use in horses in Australia in 2012 [39]. This vaccine is a subunit vaccine, which comprises the soluble form of HeV-G (HeVsG) [19,39,40,41]. The subunit HeVsG vaccine has been shown to protect against NiV in cats [42], ferrets [43], and non-human primates [44], but not in pigs [45]. The ability of HeVsG to protect against NiV infection in some animal models warrants the evaluation of a vaccine that contains a soluble form of NiV glycoprotein G.

The role of antibodies in protective immunization against NiV infection has been widely reported, however there is limited information on NiV-specific T-cell-mediated immunity. Consequently, it is of interest to better understand the role of T cells in vaccine-induced protection against NiV. This, in turn, will aide in the development of new and improved vaccine candidates.

In this study, we investigated recombinant Modified Vaccinia virus Ankara (MVA) for delivery of NiV-G antigens [46]. We constructed two MVA–NiV-G vaccine candidates to express full-length NiV-G or the soluble form NiVsG. The candidate vaccines were genetically stable and efficiently replicated in primary chicken embryo fibroblasts, a cell culture system used for manufacturing of MVA vaccines. Importantly, vaccination of mice lacking the interferon alpha/beta receptor (IFNAR−/−) elicited readily detectable NiV-G-specific CD8 and CD4 T cells. We identified a potential H2-b-restricted epitope in the NiV-G that stimulated antigen-specific CD8 T cells and a potential H2-IAb-restricted epitope that stimulated antigen-specific CD4 T cells. Interestingly, in comparison with full-length NiV-G, the soluble antigen NiVsG induced significantly stronger epitope-specific T cell responses. Our work will be relevant for future studies characterizing NiV-G-specific T cell responses in NiV preclinical models.

## 2. Materials and Methods

### 2.1. Mice

IFNAR−/− mice [47] backcrossed more than 20-fold on the C57BL/6 background (were bred under specified-pathogen-free (SPF) conditions, housed in isolated cage units (IsoCage, Tecniplast, Hohenpeißenberg, Germany) and had access to food and water ad libitum. All experiments were approved by the Government of Upper Bavaria, Munich Germany and were performed in compliance with the German Animal Welfare Act (55.2Vet-2532.Vet_02-17-93, 09.01.2017).

### 2.2. Cells

Primary chicken embryo fibroblasts (CEF) were isolated from 10-day-old SPF chicken embryos (VALO, Cuxhaven, Germany) and grown in Minimum Essential Medium (MEM) (Sigma-Aldrich, Taufkirchen, Germany) supplemented with 10% heat-inactivated fetal bovine serum (FBS) (Sigma-Aldrich), 1% Penicillin–Streptomycin (Sigma-Aldrich), and 1% MEM nonessential amino acid solution (Sigma-Aldrich). Human HeLa cells (ATCC CCL-2) were maintained in MEM supplemented with 10% heat-inactivated FBS (Sigma-Aldrich) and the above antibiotics. DF-1 cells (ATCC CRL-12203) were grown in VLE Dulbecco’s Modified Eagle’s Medium (DMEM) (Merck, Darmstadt, Germany) supplemented with 10% heat-inactivated FBS (Sigma-Aldrich), 1% Penicillin–Streptomycin (Sigma-Aldrich), 1% MEM nonessential amino acid solution (Sigma-Aldrich), and 1% HEPES solution (Sigma-Aldrich). Cells were maintained at 37 °C in a humidified 5% CO_2_ atmosphere.

### 2.3. Plasmid Constructions

The cDNA that encoded for the entire 602 amino acid sequence of the NiV-G protein (Nipah virus isolate UMMC1, GenBank accession number AY029767.1) was modified in silico by introducing silent codon alterations to remove termination signals of vaccinia virus early transcription (TTTTTNT) and G/C nucleotide runs. For construction of the soluble form of NiV-G protein (NiVsG), the cytoplasmic and transmembrane domains were deleted and an internal leader sequence and amino acid linker sequences were added as described previously [40,48]. For vaccinia virus (VACV)-specific transcriptional regulation, we placed the NiVsG gene sequences under control of the synthetic VACV early/late promoter (PmH5) [49] and used the strong natural early VACV promoter Pvgf [50,51,52] for expression of NiV-G sequences. The cDNAs encoding for NiV-G or NiVsG including the cleavage sites for the restriction endonucleases *Xho*l and *Apa*l were generated by DNA synthesis (GeneWiz, Leipzig, Germany). cDNA sequences were cloned into the MVA transfer plasmid pLW-73 [53], which directs insertion of heterologous sequences to a site between the open reading frames (ORF) of the essential viral genes, *MVA069R* and *MVA070L*.

### 2.4. Generation of Recombinant Viruses

Recombinant MVA viruses expressing the NiVsG and NiV-G proteins were generated as described previously [54,55]. To summarize, CEF cells at 80–90% confluence were infected with nonrecombinant MVA (clonal isolate MVA F6) at a multiplicity of infection (MOI) of 0.05 and transfected with vector plasmid DNA containing NiVsG or NiV-G gene sequences using X-tremeGENE™ HP DNA Transfection Reagent (Roche Diagnostics, Penzberg, Germany). After 48 h incubation, cells were harvested, and the recombinant viruses MVA–NiVsG and MVA–NiV-G were clonally isolated by screening for co-expression of the fluorescent protein marker GFP and plaque passaging several times. Resultant vector virus isolates were quality controlled using standard protocols [54]. Polymerase chain reaction (PCR) analysis of genomic viral DNA served to confirm the genetic identity and genomic stability of the MVA vector viruses. The replicative capacity of the recombinant MVA–NiV viruses compared with nonrecombinant MVA was tested by multi-step growth experiments in CEF and human HeLa cells. To generate vaccine preparations, recombinant MVA–NiV viruses were amplified in CEF, purified by ultracentrifugation through 36% sucrose cushions, and reconstituted in 10 mM Tris-HCl buffer, pH 9.0, to make stock preparations [54].

### 2.5. Western Blot Analysis of Recombinant Proteins

CEF and HeLa cells were infected with recombinant MVA–NiVsG and MVA–NiV-G viruses at a MOI of 5. Cells infected with MVA (MOI 5) and inoculation medium alone (mock) were used as controls. Cell lysates were prepared or culture supernatants were collected at various time points after infection and stored at −80 °C. Cell-associated and secreted proteins were resolved by sodium dodecyl sulfate (SDS)-polyacrylamide (10%) gel electrophoresis (SDS-PAGE), and proteins were transferred onto nitrocellulose membranes by wet electroblotting. To investigate the glycosylation pattern, proteins were pretreated using the Protein Deglycosylation Mix II kit (New England Biolabs, Ipswich, MA, USA) according to the manufacturer’s instructions prior to SDS PAGE. Membranes were blocked with blocking buffer, which consisted of PBS containing 5% non-fat dried milk powder (Carl Roth, Karlsruhe, Germany) and 0.1% *v/v* Tween 20 (Sigma-Aldrich) for one hour at room temperature. Membranes were then probed with the primary antibody (polyclonal mouse anti-NiV-G (1:2000) or polyclonal rabbit anti-NiV-G (1:5000)) diluted in blocking buffer overnight at 4 °C. Membranes were washed three times with PBS containing 0.1% Tween 20 (PBS/T) and probed with goat anti-mouse IgG conjugated to horseradish peroxidase (HRP) (1:5000; Agilent Dako, Glostrup, Denmark) or goat anti-rabbit IgG HRP (1:5000; Cell Signaling Technology, Leiden, The Netherlands). Membranes were washed again with PBS/T and were developed using SuperSignal^®^ West Dura Extended Duration substrate (Thermo Fisher Scientific, Planegg, Gemany). Blots were visualized using a MicroChemi 4.2 imager (DNR Bio-Imaging Systems, Neve Yamin, Israel).

### 2.6. Immunofluorescence

Confluent monolayers of HeLa cells were infected with recombinant MVA–NiVsG or MVA–NiV-G viruses at MOI 0.05. Controls included MVA (MOI 0.05) and inoculation medium alone (mock). After infection, cells were incubated for 16 h in a 37 °C incubator. Cells were fixed with 4% paraformaldehyde (PFA) (Sigma-Aldrich) and on some occasions, permeabilized with 0.5% Triton X-100 (Sigma-Aldrich). Cells were blocked in blocking buffer containing 1% bovine serum albumin (BSA) (Sigma-Aldrich). Cells were stained with polyclonal rabbit anti-NiV-G diluted 1:10,000 in PBS containing 0.5% BSA (PBS/BSA). Cells were washed with PBS and stained with the secondary antibody goat anti-mouse IgG Alexa Fluor (AF) 568 (1:1000; Thermo Fisher Scientific) diluted in PBS/BSA buffer. Fluorescence was visualized using the Keyence BZ-X710 microscope (Keyence, Osaka, Japan).

### 2.7. Immunization Experiments in Mice

Mice were immunized with 10^8^ PFU in 50 μL vaccine buffer (10 mM Tris and 140 mM NaCl, pH 7.4) of recombinant MVA–NiVsG, recombinant MVA–NiV-G or MVA or vaccine buffer as a mock vaccine control via the intraperitoneal or intramuscular routes. Mice received either one (prime) or two (prime-boost) immunizations over a 21 day interval. For T cell analysis, mice were euthanized 8 days after immunization. Blood was collected on days 0, 18, and 31. Coagulated blood was centrifuged at 1300× *g* for 5 min in MiniCollect vials (Greiner Bio-One, Alphen aan den Rijn, The Netherlands) in order to separate serum, which was subsequently stored at −20 °C until further use.

### 2.8. Quantification of Total Antigen-Specific IgG Antibodies

Antigen-specific IgG responses induced by immunization with the vaccine candidates were analyzed by enzyme-linked immunosorbent assay (ELISA) using purified soluble recombinant NiV glycoprotein G expressed in mammalian HEK293 cells. Flat bottom 96-well ELISA plates (Nunc™ MaxiSorp™ Plates, Thermo Scientific) were coated with 50 ng/well recombinant protein (100 µL volume) and incubated overnight at 4 °C. Plates were washed three times with 200 µL/well PBS/T. Plates were blocked with blocking buffer containing 1% bovine serum albumin (Sigma-Aldrich) and 0.15 M sucrose (Sigma-Aldrich) in PBS for 1 h at 37 °C. Plates were then washed with PBS/T as described above. Sera were serially diluted in PBS containing 1% BSA (PBS/BSA) three-fold down the plate, starting at a dilution of 1:100 (100 µL volume/well) and incubated for 1 h at 37 °C. After washing, plates were incubated with 100 µL/well goat anti-mouse IgG conjugated HRP (1:2000; Agilent Dako, Denmark) diluted in PBS/BSA for 1 h at 37 °C. Plates were then washed with PBS/T as described earlier. Then, 100 µL/well 3,3′, 5,5′-Tetramethylbenzidine (TMB) Liquid Substrate System for ELISA (Sigma-Aldrich) was added, and plates were incubated until a color change was observed. The reaction was stopped by the addition of 100 µL/well Stop Reagent for TMB Substrate (450 nm, Sigma-Aldrich). The absorbance was measured on an ELISA plate reader at 450 nm with a 620 nm reference wavelength. Total IgG titers were calculated from the inflection point of the titration curve as logarithms of the reciprocal.

### 2.9. Generation of Synthetic Peptides, Design of Peptide Pools, and Peptide Prediction

The protein sequence for NiV-G was obtained from the UniProt database (ID: Q9IH62). Using an in silico approach, we identified 130 individual synthetic peptides that spanned the external domain NiV-G protein, starting from the third amino acid of the N-terminal side to the C terminus (amino acids 73–602). Our peptide library consisted of 15mer peptides that overlapped by 11mer. All peptides were synthesized by Thermo Fisher Scientific as crude material (<50% purity) on a 1–4 mg scale. The two-dimensional peptide pool matrix system was used for screening [56,57]. Briefly, peptides were organized into two-dimensional matrix peptide pools (H1–H10 and V1–V11) containing 11–13 peptides. For mapping of potential CD8 T cell epitopes in positive 15mer peptides, every possible sequence of peptides 8–11mer in length was determined. Theoretical peptides were then analyzed for binding to the mouse major histocompatibility complex (MHC) class I allele H2-b using the SYFPEITHI database. The peptides of each amino acid sequence length were synthesized and tested. For identification of CD4 T cell epitopes, MHC class II binding predictions were performed on 15mer peptides found within positive peptide pools identified by IFN-γ Enzyme-Linked Immunospot assay (ELISPOT). Using the MHC Class II Binding, T Cell Epitope Prediction resource of the Immune Epitope Database (IEDB, https://www.iedb.org/), peptides restricted to mouse MHC class II allele H2-IAb were analyzed using “IEDB Recommended” prediction method [58]. The most promising candidates were then chosen for further experimental epitope prediction studies. All peptides were dissolved to a concentration of 2 mg/mL in PBS, aliquoted, and stored at −20 °C until use.

### 2.10. T Cell Analysis

#### 2.10.1. Enzyme-Linked Immunospot assay (ELISPOT)

T cell analysis by ELISPOT was performed as described previously [55]. Briefly, spleens were collected from mice 8 days after the final immunization. Single-cell suspensions were prepared by teasing spleens through a 70 µm cell strainer (Falcon^®^ Corning, Corning, NY, USA). Red blood cells (RBC) were removed using Red Cell Lysis Buffer (Sigma-Aldrich), and cells were washed and resuspended in RPMI-10, which consisted of RPMI-1640 (Sigma-Aldrich) containing 10% heat-inactivated FBS (Sigma-Aldrich) and 1% Penicillin–Streptomycin (Sigma-Aldrich). For experiments that required CD4 and CD8 T cell purification, splenocytes were incubated with mouse CD4 and CD8 MicroBeads (Miltenyi Biotec, Bergisch Gladbach, Germany) and processed by negative selection using the QuadroMACS Separator (Miltenyi Biotec).

IFN-γ-producing cells were measured by IFN-γ ELISPOT assay using the Mouse IFN-γ ELISpotPLUS kit (Mabtech, Stockholm, Sweden) as described in the manufacturer’s protocol. In summary, 2 × 10^5^ splenocytes were seeded onto 96-well flat bottom plates (100 µL/well) (Sarstedt, Nümbrecht, Germany), and 100 µL/well peptide pools, subpools, or individual peptides were added (each peptide diluted to 2 µg/mL in RPMI-10). After mixing, the splenocyte/peptide mixtures were transferred onto plates precoated with IFN-γ detection antibody and incubated for 48 h at 37 °C. Nonstimulated cells were used as a mock control, and the positive controls cultures were treated with phorbol myristate acetate (PMA) and ionomycin (both from Sigma-Aldrich) or vaccinia virus (VACV)-specific CD8 T cell epitope, B8R_20–27_ (TSYKFESV) [59]. After incubating, plates were processed as described in the kit manufacturer’s protocol (Mabtech). Spots were counted and analyzed using the Automated ELISPOT plate reader (A. EL. VIS Eli.Scan and A. EL. VIS ELISPOT Analysis Software, Hannover, Germany).

#### 2.10.2. Intracellular Cytokine Staining and Flow Cytometry

Splenocytes were diluted to 1 × 10^7^ cells/mL in RPMI-10, and 100 µL/well (1 × 10^6^ cells) was added onto a 96-well U-bottom plate. Then, 100 µL/well peptide diluted to 16 µg/mL in RPMI-10 was added to give a final peptide concentration of 8 µg/mL. The VACV CD8 T cell epitope B8R_20–27_ (final concentration of 8 µg/mL) was used as a positive control along with PMA (10 ng/mL) plus ionomycin (500 ng/mL). PBS diluted in RPMI-10 was used as a mock stimulated control. After plating, cells were incubated for 2 h at 37 °C. Then, 20 µL/well 10x Brefeldin A, a Golgi inhibitor that was prepared by diluting 1000X Brefeldin A (Biolegend, San Diego, CA, USA) in RMPI-10, was added to give a final dilution of 1x Brefeldin A. Cells were then incubated for an additional 4 h at 37 °C. After incubating, plates were centrifuged (500 *g* for 3 min), and the supernatant was removed. Cells were washed once with 200 µL/well FACS buffer (MACSQuant Running Buffer with 2% FBS). Extracellular staining was performed with the following antibodies diluted in FACS buffer: anti-mouse CD3 phycoerithrin (PE)-Cy7 (clone 17A2, 1:100, Biolegend), anti-mouse CD4 Brilliant Violet 421 (clone GK1.5, 1:600, Biolegend), anti-mouse CD8α Alexa Fluor 488 (clone 53-6.8, 1:300, Biolegend), and purified CD16/CD32 (Fc block; clone 93, 1:500, Biolegend). Cells were stained in 50 µL/well for 30 min on ice in the dark, then washed once with 200 µL/well FACS buffer and then twice with 200 µL/well PBS. Cells were then stained with 100 µL/well of the fixable dead cell viability dye Zombie Aqua (1:800, Biolegend) diluted in PBS for 30 min on ice in the dark. Cells were washed twice with 200 µL/well PBS and fixed with 100 µL/well of Fixation Buffer (Biolegend) for 20 min at room temperature in the dark. Cells were washed twice with 200 µL/well PBS and resuspended in 200 µL/well FACS buffer. Plates were stored overnight at 4 °C, protected from light. The following day, cells were permeabilized by washing three times with 200 µL/well 1× Perm Wash buffer, which contained Intracellular Staining Permeabilization Wash Buffer (10×) (Biolegend) diluted to 1× with distilled water. Cells were stained intracellularly in 100 µL/well of anti-mouse IFN-γ (clone XMG1.2, 1:200, Biolegend) plus TNF-α (clone MP6-XT22, 1:200, Biolegend) diluted in 1X Perm Wash buffer for 30 min at room temperature, protected from light. Cells were then washed three times with 200 µL/well 1X Perm Wash and then resuspended in 300 µL/well FACS buffer. Samples were filtered through 50 µm nylon mesh (Sefar Pty Ltd., Huntingwood, NSW, Australia) into 5 mL round bottom FACS tubes (Sarstedt). Single-color controls were prepared for each FACS analysis using OneComp eBeads™ Compensation Beads (eBioscience, Thermo Fisher Scientific) for fluorophore-conjugated antibodies and cells for the viability dye Zombie Aqua. Data acquisition was performed by MACSQuant VYB Flow Analyser (Miltenyi Biotec), and data was analyzed using FlowJo (FlowJo LLC, BD Life Sciences, Ashland, OR, USA).

### 2.11. Statistical Analysis

Data were analyzed using GraphPad Prism version 5.0 (GraphPad Software Inc., San Diego, CA, USA) and were expressed as mean ± standard error of the mean (SEM). Statistical analysis was performed using the unpaired, two-tailed *t*-test to compare two groups and one-way ANOVA to compare three or more groups. The threshold for statistical significance was *p* < 0.05.

## 3. Results

### 3.1. Generation and Characterisation of MVA Vector Vaccines Delivering NiV-G Antigens

To generate the recombinant MVA viruses delivering NiV-G antigens, we used the gene from NiV Malaysia (isolate UMMC1, GenBank accession number AY029767.1) and generated codon-optimized gene sequences encoding a full-length glycoprotein G (NiV-G) or a soluble external domain G protein (NiVsG). These synthetic gene sequences were placed under the transcriptional control of the vaccinia virus-specific promoters Pvgf or PmH5 and introduced into the MVA genome by homologous recombination targeting the intergenic site between the essential MVA genes *069* and *070L*. The clonal isolation of the recombinant viruses MVA–NiVsG and MVA–NiV-G was facilitated by the co-production of the green fluorescent reporter protein (GFP), as previously described [54]. The final recombinant viruses containing the NiV-G gene sequences (MVA–NiV-G and MVA–NiVsG) were obtained after removal of the GFP reporter gene by intragenomic homologous recombination (Figure S1A, marker gene deletion). To verify the identity of the desired modification, we performed the standard quality control experiments as described previously [54]. PCR analysis of viral genomic DNA confirmed the proper insertion of the recombinant gene sequences at the target site in the genome of MVA (Figure S1B–E) and the genetic characteristics and stability of the recombinant MVA viruses. We assessed the growth behavior of the recombinant viruses MVA–NiVsG and MVA–NiV-G in multi-step growth analyses in human HeLa cells and primary CEF, which are routinely used for amplification of recombinant MVA in vaccine manufacturing (Figure S1F). MVA–NiVsG and MVA–NiV-G efficiently replicated in CEF and demonstrated an increase of infectivity titers that was comparable to that obtained with nonrecombinant MVA. However, MVA–NiV-G and MVA–NiVsG did not productively grow in human HeLa cells, confirming that they had retained the characteristic replication deficiency of MVA in cells of mammalian origin. These findings corroborated the expected MVA phenotype and confirmed that the recombinant viruses could be handled under laboratory conditions of biosafety level 1. Of note, we originally generated another recombinant MVA virus using the synthetic early–late promoter PmH5 for transcriptional control of recombinant gene expression and production of the full-length NiV-G protein. However, upon growth testing, this candidate virus failed to reach levels of infectious progeny in CEF to be eligible for large-scale amplification as needed for vaccine production processes.

### 3.2. Characterisation of Recombinant NiV-G Proteins

**Figure 1:**
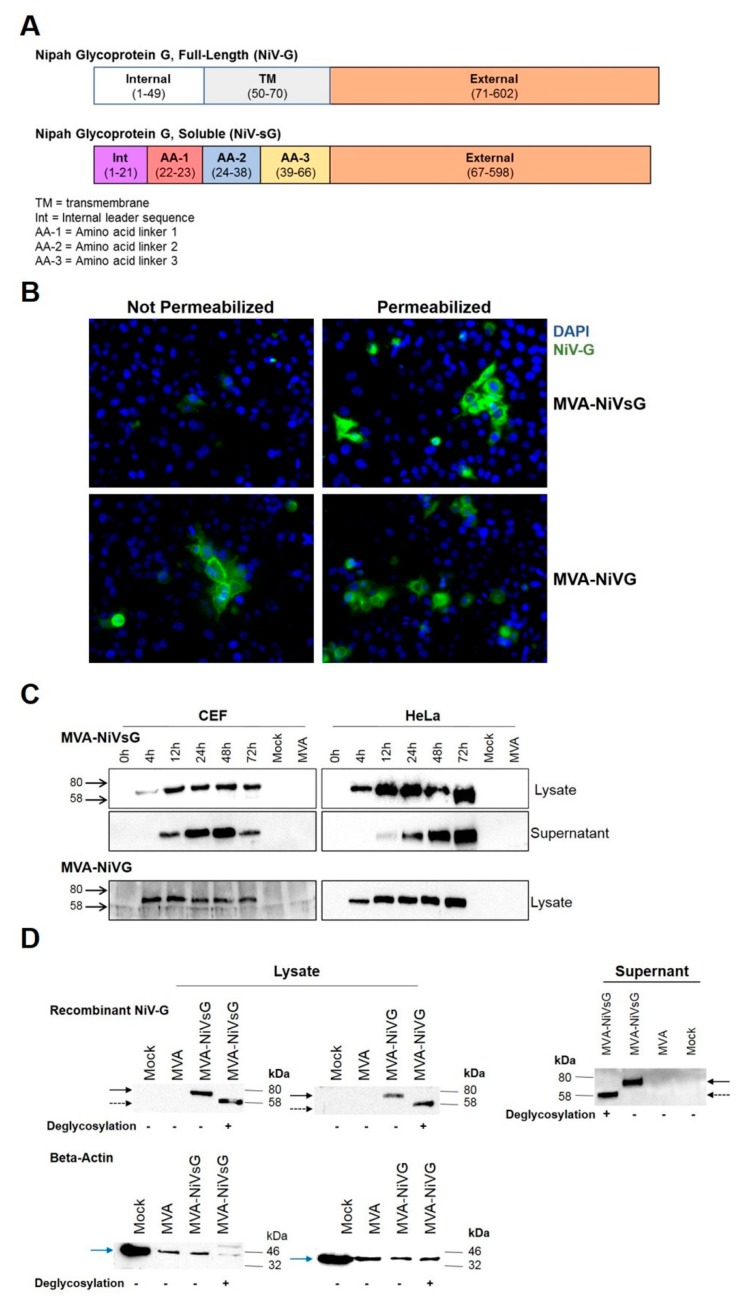
Analysis of recombinant Nipah virus full-length glycoprotein G (NiV-G) protein produced by cells infected with MVA–NiVsG and MVA–NiV-G. (**A**) Schematic diagram of recombinant NiV full-length glycoprotein G (NiV-G) and NiV soluble glycoprotein G (NiVsG). Colored rectangles represent individual protein domains, and bracketed text displays the start and end of amino acid sequences of each domain. (**B**) Immunofluorescence staining of cells infected with MVA–NiVsG and MVA–NiV-G. HeLa cells were infected at MOI 0.05 with the above viruses for 16 h. MVA and mock infected HeLa cells were used as controls. Fixed permeabilized and fixed nonpermeabilized cells were immunostained with rabbit polyclonal antibody for NiV-G and the secondary antibody anti-rabbit Alexa Fluor 488. Nuclei were stained with DAPI solution. Panel shows representative pictures of fixed/permeabilized and fixed/nonpermeabilized infected HeLa cells at 40× magnification. (**C**) Western blot analysis of recombinant NiV-G proteins produced by chicken embryo fibroblasts (CEF) and HeLa cells infected with MVA–NiVsG and MVA–NiV-G. Lysates and culture supernatants were collected from cell cultures infected at MOI 5 with the above viruses, wild-type MVA or noninfected controls (mock). Samples were collected at indicated hours post-infection. Cell lysates and proteins were tested by immunoblotting using a NiV-G-specific polyclonal mouse antibody. Protein bands corresponding to the expected molecular weights of recombinant NiV-G and NiVsG protein (~65−70 kDa) are indicated. (**D**) Western Blot analysis of recombinant proteins produced by DF-1 cells infected with MVA–NiVsG and MVA–NiV-G at MOI 5 for 36 h. MVA and mock infected cells were used as controls. Cell lysates and culture supernatants were incubated with (+) or without (−) enzymes to deglycosylate proteins, analyzed by SDS-PAGE, and immunoblotted with a rabbit polyclonal antibody for NiV-G. Beta-actin was used as a loading control for lysates. Solid black arrow represents glycosylated recombinant NiV-G and NiVsG protein (~65–70 kDa), and dashed black arrow represents deglycosylated recombinant NiV-G and NiVsG protein (~58 kDa). Blue solid arrow represents beta-actin (~40 kDa). MVA: modified Vaccinia virus Ankara.

Our vaccine candidates, MVA–NiVsG and MVA–NiV-G, should produce recombinant NiV glycoprotein G in its full-length form (NiV-G) and, in parallel, the soluble form (NiVsG). NiV-G is a 602 amino acid long protein consisting of an N-terminal internal domain, a transmembrane domain, and a C-terminal external domain (Figure 1A). For NiVsG protein, an internal leader sequence and three amino acid linkers have replaced the internal and transmembrane domains (Figure 1A), as described previously [19,48]. We assessed the correct expression and studied the cellular localization of the NiV-G and NiVsG by immunofluorescence microscopy of MVA–NiV-infected cells immunolabeled with anti-NiV-G antibody, followed by a fluorescently labelled secondary antibody. Cell nuclei were stained with DAPI (300 nM). As anticipated, we observed different patterns of green fluorescence, with varying cellular localizations depending on the MVA–NiV construct. Green fluorescence, specific for NiV-G, was identified in permeabilized and nonpermeabilized cells infected with MVA–NiV-G (Figure 1B), but not in MVA-infected or mock control cells. This data confirmed that the recombinant full-length NiV-G protein encoded by MVA–NiV-G was indeed anchored on the cell surface. In contrast, the NiV-G-specific staining in cells infected with MVA–NiVsG virus appeared to be predominantly located within the cells and was readily detected after permeabilization. As anticipated, we failed to detect NiVsG in considerable amounts without permeabilization, confirming that NiVsG was not expressed on the cell surface (Figure 1B).

To further investigate the synthesis of NiV-G proteins after infection with MVA–NiVsG and MVA–NiV-G, respectively, total proteins from infected CEF and HeLa cell cultures were analyzed by Western blot using a NiV-G-specific antibody. Total cell lysates or culture supernatants obtained from CEF and HeLa cultures infected with recombinant MVA virus were separated by SDS-PAGE and immunoblotted. We specifically detected a protein with an estimated molecular mass of approximately 72–75 kDa in lysates from CEF cells and HeLa cells infected either with MVA–NiV-G or MVA–NiVsG (Figure 1C). In the cell lysates, the glycoprotein was first detectable at 4 h post-infection. The amount of protein increased over time, resulting in a prominent band at 16 h post-infection, which was maintained throughout the 72 h time course. To monitor the release of recombinant NiV-G protein from MVA–NiVsG infected cells, we also analyzed the supernatants of infected CEF and HeLa cell cultures. A protein with a molecular mass of approximately 72–75 kDa sized band was observed in supernatants from MVA–NiVsG-infected cells (Figure 1C), indicating that infected cells were secreting the soluble NiV-G protein. Secreted NiV-G protein was first observed at 16 h post-infection in the supernatants of MVA–NiVsG-infected cultures and became especially prominent by 24 h post-infection. The levels of secreted NiV-G seemed to decline by 72 h post-infection. We did not however detect NiV-G protein in supernatants of MVA–NiV-G-infected cells.

Since the NiVsG gene encoding sequences were modified to result in the secreted soluble version (sG), we performed additional Western blot experiments to determine whether the protein still maintained glycosylation sites comparable to wild-type NiV-G. Lysates and supernatants obtained from cultures of DF-1 cells infected with MVA–NiVsG were treated with enzymes that deglycosylate proteins and analyzed by western blotting. Enzyme treatment resulted in a reduction in the molecular mass of recombinant NiVsG protein from 70–75 kDa to 58–60 kDa (Figure 1D), matching the expected size of unmodified G protein. This suggested that recombinant NiVsG has retained the normal glycosylation pattern of wild-type NiV-G.

### 3.3. Antibody Responses in Vaccinated IFNAR−/− Mice

**Figure 2:**
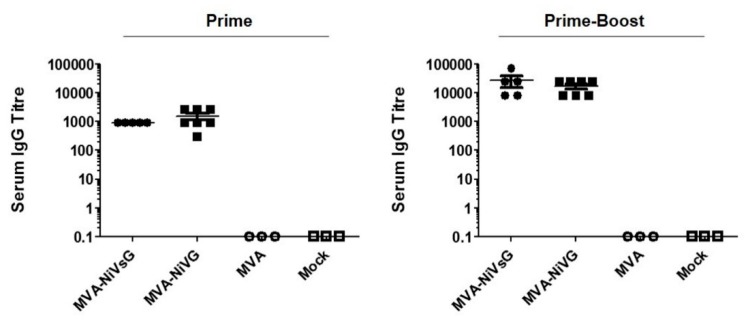
Antigen-specific humoral immunity induced by treatment with recombinant MVA vaccine candidates expressing NiV-G. IFNAR−/− mice were immunized and boosted 21 days later with 10^8^ PFU of MVA–NiVsG or MVA–NiV-G via the intramuscular (i.m.) route (*n* = 3–7 per group). Mice inoculated with MVA or saline (mock) were used as controls. Sera collected 18 days after the first immunization (Prime) and 10 days (Prime-Boost) after the last immunization were analyzed for NiV-G-specific IgG titers by ELISA. Graphs show the antigen-specific serum IgG titer.

To assess the immunogenicity of the recombinant MVA–NiV-G/NiVsG candidate vaccines, we vaccinated IFNAR−/− mice with 10^8^ PFU via the intramuscular route at days 0 and 21. Serum samples were tested for NiV-G-binding IgG antibodies by ELISA 18 days after the first immunization (Prime) and 10 days after the second immunization (Prime-Boost) (Figure 2). Even a single application of the MVA–NiV-G vaccines induced abundant levels of NiV-G-specific IgG antibodies in the mice. After booster immunization, all vaccinated animals produced even higher levels of circulating NiV-G-specific antibodies, with the antibody titers increasing by approximately ten-fold.

### 3.4. NiV-G Specific T Cell Immunity Induced by Immunization with Recombinant MVA Viruses Expressing NiV-G or NiV-sG

#### 3.4.1. Screening for NiV-G Epitopes Using Pools of Overlapping Peptides

**Figure 3:**
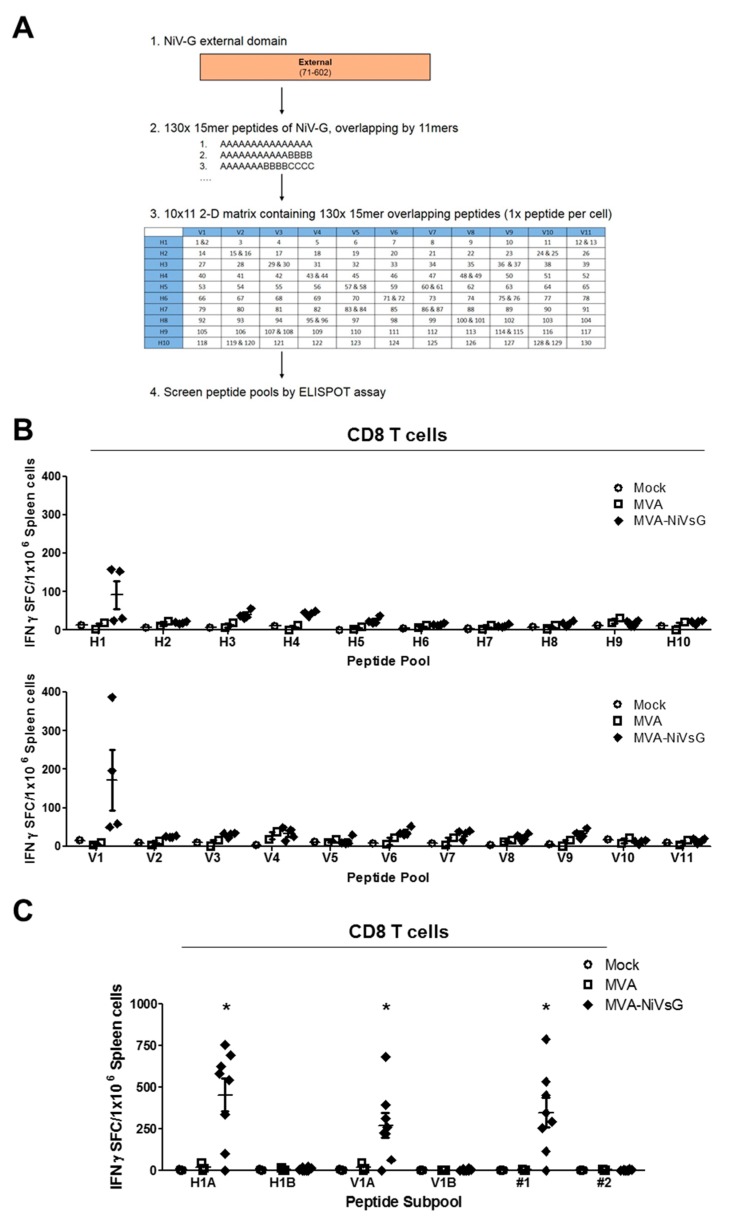
Screening for H2-b-restricted T cell epitopes of NiV-G protein. IFNAR−/− mice were immunized twice with 10^8^ PFU of MVA–NiVsG via the intraperitoneal (i.p.) route over a 21 day period (2–5 per group). Mice inoculated with nonrecombinant MVA (MVA) or saline (mock) were used as controls. Eight days after the booster immunization, spleens were collected and single cell suspensions were prepared. CD8 T cells were purified by magnetic bead selection and restimulated with pools of 10–13 overlapping 15mer peptides of NiV-G or subpools of positive peptide pools and analyzed by IFN-γ ELISPOT assay. (**A**) Schematic overview of peptide mapping strategy. A peptide library was generated comprising of 15mer peptides (overlapping by 11mer) that spanned the external domain of the wild-type NiV-G protein. Peptides were organized into a two-dimensional matrix of peptide pools (horizontal rows (H)1–(H)10 and vertical rows (V)1–(V)11) containing 11–13 peptides. Each cell of the matrix represents an individual 15mer peptide. (**B**) Screening for H2-b-restricted T cell epitopes of NiV-G in CD8 T cell enriched splenocytes measured by ELISPOT assay. Graphs shows IFN-γ spot-forming cells (IFN-γ SFC) of CD8 T cells stimulated with peptide pools. (**C**) Screening of subpools of positive peptide pools H1 (H1A and H1B) and V1 (V1A and V1B) and individual 15mer peptides shared between the two pools, #1 (YTRSTDNQAVIKDAL) and #2 (TDNQAVIKDALQGIQ). Graphs shows IFN-γ SFC (spot-forming counts) of stimulated CD8 T cells. Differences between groups were analyzed by one-way ANOVA. Asterisks represent statistically significant overall differences for a specific peptide subpool or individual peptide. * *p* < 0.05.

Currently, there is only limited information available on NiV-specific T cell immunity. In order to characterize the NiV-G-specific T cell response induced by our candidate vaccines, we first aimed to identify NiV-G polyprotein-specific T cell epitopes. IFNAR−/− mice on a C57BL/6 background (MHC I = H2-Db/H2-Kb (H2-b) and MHC II = H2-IAb) were immunized twice with the MVA–NiVsG candidate vaccine via the intraperitoneal (i.p.) route, and splenocytes were prepared 8 days after the final inoculation, CD4 and CD8 T cells were purified and restimulated with overlapping 15mer peptides corresponding to the NiV-G protein. We pooled overlapping peptides using a two-dimensional peptide matrix system (Figure 3A) and screened for their ability to induce IFN-γ by ELISPOT. IFN-γ production above background levels was observed after stimulation with 2 out of the 21 peptide pools tested (Figure 3B). CD8 T cells from mice immunized with MVA–NiVsG, but not the MVA and mock groups, showed elevated numbers of IFN-γ spot-forming counts (SFC) after stimulation with peptide pools V1 and H1 (Figure 3B). In the next step, the peptides within the V1 and H1 peptide pools were used to characterize the T cell epitope specificities in more detail. For this, pools V1 (V1A and V1B) and H1 (H1A and H1B) were subdivided into subpools containing 5–7 peptides each. Since two 15mer peptides were shared between the pools, #1 (G_73–87_ = YTRSTDNQAVIKDAL) and #2 (G_77–91_ = TDNQAVIKDALQGIQ), these peptides were also tested separately. For this experiment, we used an identical immunization protocol and splenic CD8 T cell purification procedure. CD8 T cells from these mice were restimulated with the above subpools and individual peptides #1 and #2. Stimulation with the subpools H1A and V1A resulted in IFN- γ production above background levels in the MVA–NiVsG group with IFN-γ SFC mean ± SEM values of 455 ± 99 and 271 ± 74 IFN-γ SFC/10^6^ splenocytes, respectively (Figure 1C). Subpools H1B and V1B, however, did not stimulate CD8 T cells in the MVA–NiVsG group. Stimulation of CD8 T cells with individual peptides #1 and #2, which were present in subpools H1A and V1A, resulted in different outcomes. IFN-γ production above the background was observed in cultures stimulated with #1 (mean = 349 ± 87 SFC/10^6^ cells), but not #2, in the MVA–NiVsG group. These data suggested that the 15mer peptide #1 contained peptide sequences that stimulated NiV-G-specific CD8 T cells.

#### 3.4.2. Identification of Potential H2-b-Restricted CD8 T Cell Epitopes of NiV-G

**Figure 4:**
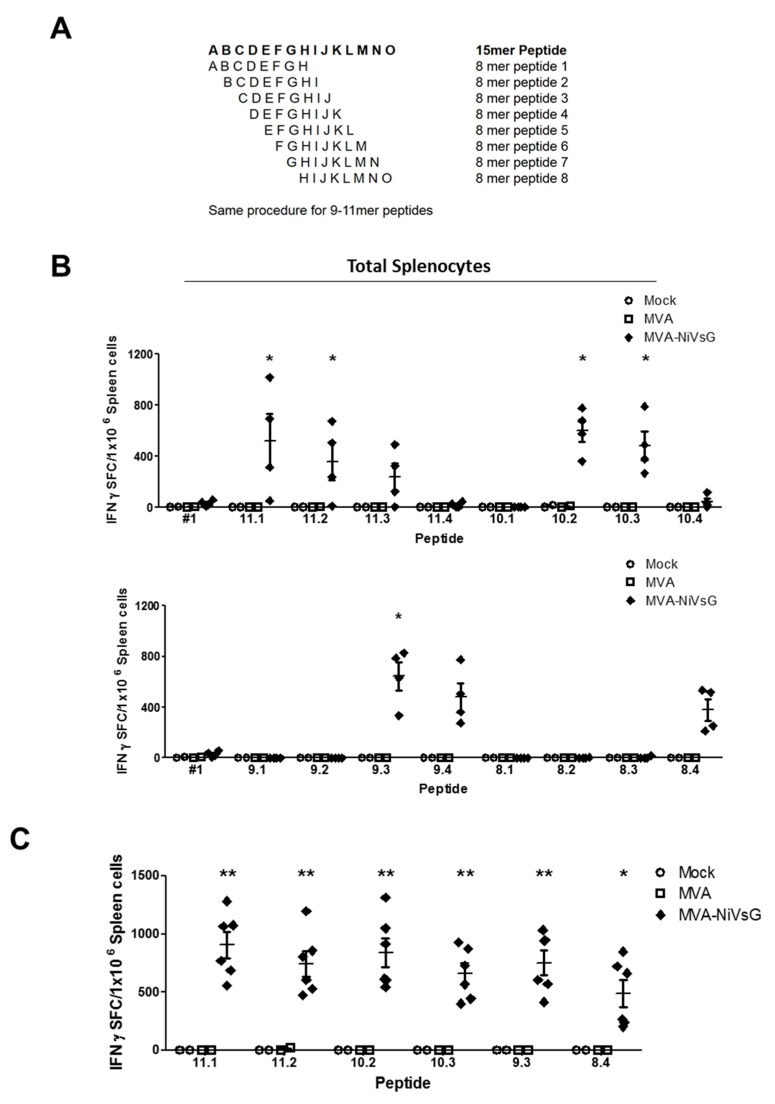
Identification of H2-b-restricted T cell epitopes in the NiV-G protein. Groups of IFNAR−/− mice (*n* = 2–4) were immunized twice with MVA–NiVsG, MVA, or saline (mock) via the i.p. or intramuscular (i.m.) routes over a 21 day period. Spleens were collected and single cells suspensions were prepared 8 days after the final immunization. Total splenocytes were restimulated and measured by ELISPOT assay. (**A**) Schematic overview of 8–11mer overlapping peptide generation. The amino acid sequence of the positive 15mer peptide, #1, served to generate peptides with every possible 8–11mer sequence. Sequences were selected based on H2-b binding prediction results obtained from the SYFPEITHI database. (**B**) Mapping of H2-b-restricted 8–11mer overlapping peptides spanning the positive 15mer peptide #1 (YTRSTDNQAVIKDAL). Graphs show IFN-γ SFC of total splenocytes from i.p. immunized mice restimulated with peptide #1 and 8–11mer overlapping peptides. (**C**) Confirmation of positive H2-b-restricted peptides of NiV-G. Graph shows IFN-γ SFC of total splenocytes from i.m. immunized mice restimulated with the six most positive 8–11mer peptides (11.1, 11.2, 10.2, 10.3, 9.3, 8.4). Differences between groups were analyzed by one-way ANOVA. Asterisks represent statistically significant overall differences for a specific peptide. * *p* < 0.05, ** *p* < 0.01.

To map the potential CD8 T cell epitope within the NiV-G protein in more detail, we dissected the 15mer peptide #1 into every possible 8–11mer sequence (Figure 4A). The sequences were then analyzed for H2-b binding computationally using the SYFPEITHI database, and the four best of each amino acid length were chosen (Table 1). To test these peptides, we immunized IFNAR−/− mice twice with MVA–NiVsG via the i.p. route and analyzed total splenocyte activation by ELISPOT assay. Of the sixteen 8–11mer overlapping peptides tested, eight resulted in the stimulation of measurable IFN-γ in the MVA–NiVsG group (Figure 4B). The positive peptides included three 11mer in length (11.1, 11.2, and 11.3), two 10mer in length (10.2 and 10.3), two 9mer in length (9.3 and 9.4), and one 8mer in length (8.4) (Figure 4B). The mean IFN-γ SFC value was lower for cells stimulated with peptide 8.4 relative to peptide 9.3. The mean values for the MVA–NiVsG group were 645 ± 112 IFN-γ SFC/10^6^ cells for peptide 9.3 and 380 ± 85 IFN-γ SFC/10^6^ for peptide 8.4.

Comparative analysis of the positive peptide sequences demonstrated that the sequence of peptide 9.3 (RSTDNQAVI) was present in all positive 10–11mer peptides (Table 1). In addition, the sequence of peptide 8.4 (STDNQAVI) was present in the eight positive peptides. To characterize the induction of IFN-γ SFC by these peptides in more detail, IFNAR−/− mice were vaccinated twice via the i.m. route, a commonly used route for human vaccinations, and in vitro stimulated splenocytes were analyzed by ELISPOT assay. For this experiment, we chose the most promising peptides of each amino acid sequence length (peptides 8.4, 9.3, 10.2, 10.3, 11.1, and 11.2). The six peptides tested significantly stimulated the activation of IFN-γ in the MVA–NiVsG group relative to the MVA and PBS controls (Figure 4C). Mean SFC values, however, did not show statistically significant variations between each individual peptide. Importantly, the mean SFC value for peptide 9.3 was again nonsignificantly elevated relative to peptide 8.4 (mean ± SEM = 663 ± 213 and 305 ± 148 IFN-γ SFC/10^6^ cells respectively). Consequently, we chose peptide 9.3 (RSTDNQAVI) (mean = 906 ± 109 IFN-γ SFC/10^6^ cells) as a potential H2-b-restricted epitope candidate of NiV-G (Table 2). The alignment of peptide 9.3 to the full sequence of NiV-G is shown in Figure S3.

#### 3.4.3. NiV-G-Specific CD8 T Cells Induced by MVA Candidate Vaccines Expressing NiV-G or NiVsG

**Figure 5:**
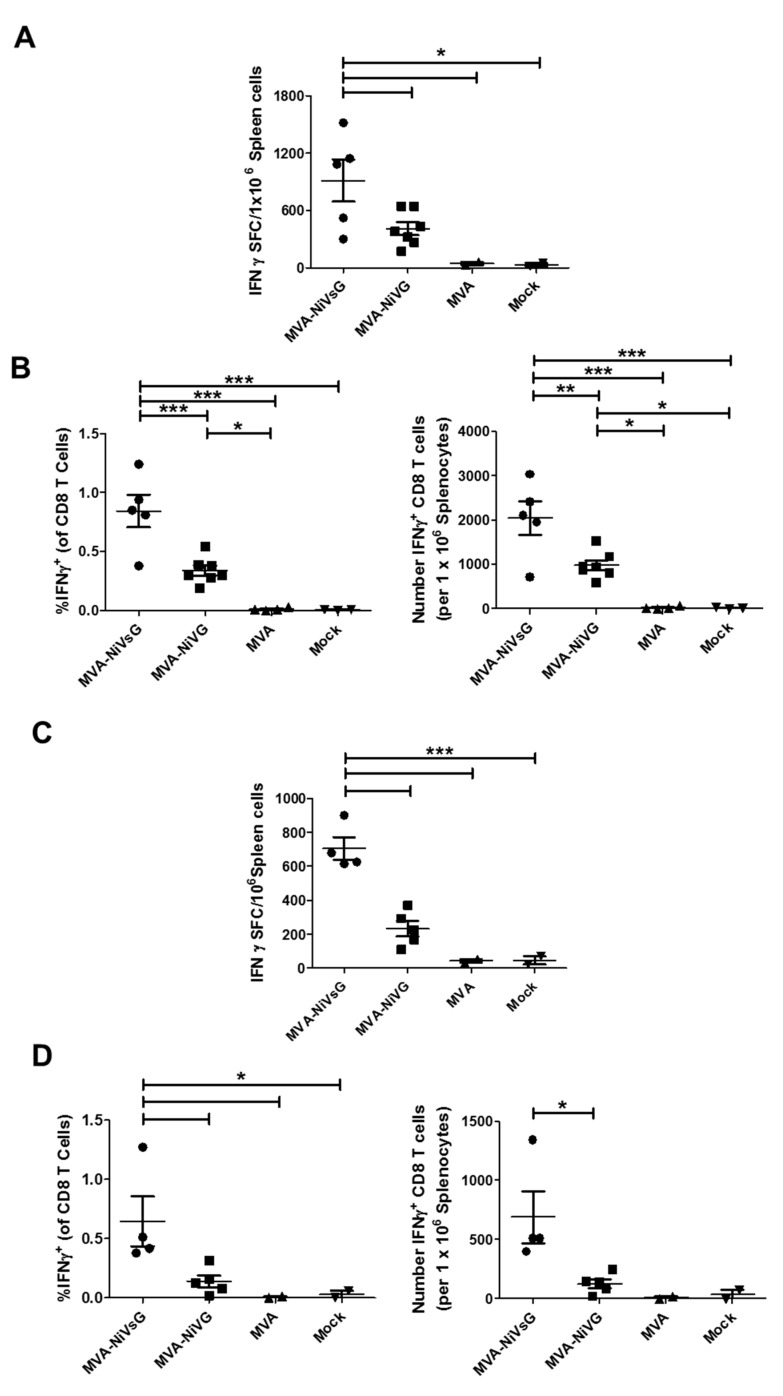
Activation of antigen-specific T cells after immunization with the recombinant MVA candidate vaccines expressing NiV-G. Groups of IFNAR−/− mice (*n* = 2–7) were immunized with MVA–NiVsG, MVA–NiV-G, MVA, or saline (mock) via the i.m. route using a prime-only or the previously described prime-boost schedule. Spleens were collected and single cell suspensions were prepared 8 days after the final immunization. Total splenocytes were restimulated with the H2-b-restricted peptide of NiV-G 9.3 (RSTDNQAVI) (Table 2) and measured by ELISPOT assay and IFN-γ ICS plus FACS analysis. (**A**,**B)** Antigen-specific CD8 T cell response induced by prime-boost immunization. (**A**) IFN-γ SFC for stimulated splenocytes measured by ELISPOT assay. (**B**) IFN-γ production by stimulated splenic CD8 T cell measured by ICS and FACS analysis. Graphs show frequency and absolute number (per 10^6^ splenocytes) of antigen-specific IFN-γ+ CD8 T cells. (**C**,**D)** Antigen-specific CD8 T cell response induced by prime immunization. (**C**) IFN-γ SFC for stimulated splenocytes measured by ELISPOT assay. (**D**) Frequency and absolute number (per 10^6^ splenocytes) of antigen-specific CD8 T cells measured by IFN-γ ICS plus FACS analysis. Differences between individual groups were analyzed by one-way ANOVA and Tukey post-hoc test. Asterisks represent statistically significant differences between two groups. * *p* < 0.05, ** *p* < 0.01, *** *p* < 0.001.

To comparatively evaluate the activation of NiV-G-specific immunity after i.m. vaccination with MVA–NiVsG and MVA–NiV-G using a standard dose of 10^8^ PFU, we stimulated splenocytes with peptide 9.3 (Table 2) and analyzed cytokine production by IFN-γ ELISPOT assay and IFN-γ plus TNF-α ICS. Comparisons between the two candidate vaccines showed that the mean SFC values were significantly higher for the MVA–NiVsG group relative to the MVA–NiV-G group (Figure 5A and Figure S2A). The means of the MVA–NiVsG group were 914 ± 221 IFN-γ SFC/10^6^ cells, and the means of the MVA–NiV-G group were 410 ± 68 IFN-γ SFC/10^6^ cells (Figure 3C).

IFN-γ ICS data showed that both the frequency and absolute number of IFN-γ+ CD8 T cells were significantly higher than the control background levels (Figure 5B). Our peptides did not induce IFN-γ production by CD4 T cells (Figure S2B), indicating that they indeed stimulated antigen-specific CD8 T cells only. When we compared our two candidate vaccines, we found that the percentage and absolute number of IFN-γ+ CD8 T cells was significantly higher in the MVA–NiVsG group. The mean frequencies of IFN-γ+ CD8 T cells were 0.84% ± 14% for the MVA–NiVsG group and 0.33% ± 0.04% for the MVA–NiV-G group. Mean absolute number of IFN-γ+ CD8 T cells were in the range of 2040 ± 380 cells/10^6^ splenocytes and 978 ± 112 cells/10^6^ splenocytes for the MVA–NiVsG and MVA–NiV-G groups, respectively. Taken together, our ELISPOT and IFN-γ ICS data indicate that MVA–NiVsG activates higher numbers of peptide-specific CD8 T cells than MVA–NiV-G. When we analyzed for coproduction of IFN-γ and TNF-α, we found that the majority of NiV-G peptide-specific CD8 T cells from MVA–NiVsG and MVA–NiVG immunized mice were double positive for the cytokines (IFN-γ + TNF-α+) (Figure S4A–C).

After showing that prime-boost immunization with our two vaccine candidates generated robust NiV-G-specific CD8 T cell responses, we tested their immunogenicity after a single vaccination. IFNAR−/− mice were vaccinated once with MVA–NiVsG, MVA–NiV-G, MVA, or mock via the i.m. route and analyzed by ELISPOT and IFN-γ + TNF-α ICS as described before. Our ELISPOT results overall showed elevated SFC counts relative to the background levels of our MVA and mock controls (Figure 5C). Statistically significant differences between the MVA–NiVsG and MVA–NiV-G were detected, with mean SFC values of 704 ± 67 IFN-γ SFC/10^6^ cells and 233 ± 46 IFN-γ SFC/10^6^ for them, respectively. FACS analysis showed that the frequency and absolute number of IFN-γ+ CD8 T cells was higher in the experimental groups relative to the MVA and mock controls (Figure 5D). The mean percentage of IFN-γ + CD8 T cells was 0.14% ± 0.049% for the MVA–NIV-G groups, whereas for the MVA–NiVsG group, the mean percentage range for the peptides was 0.65% ± 0.21%. While MVA–NiV-G prime immunization induced a low frequency of IFN-γ+ cells, the MVA–NiVsG group showed IFN-γ+TNF-α+ secreting T cells after a single vaccination (Figure S4D–F), although percentages were lower than what was observed after prime-boost immunization (Figure S4A–C). This indicates that prime immunization with MVA–NiVsG was sufficient to generate a sizeable population of polyfunctional antigen-specific CD8 T cells.

#### 3.4.4. Identification of Potential H2-IAb-Restricted CD4 T Cell Epitopes of NiV-G

**Figure 6:**
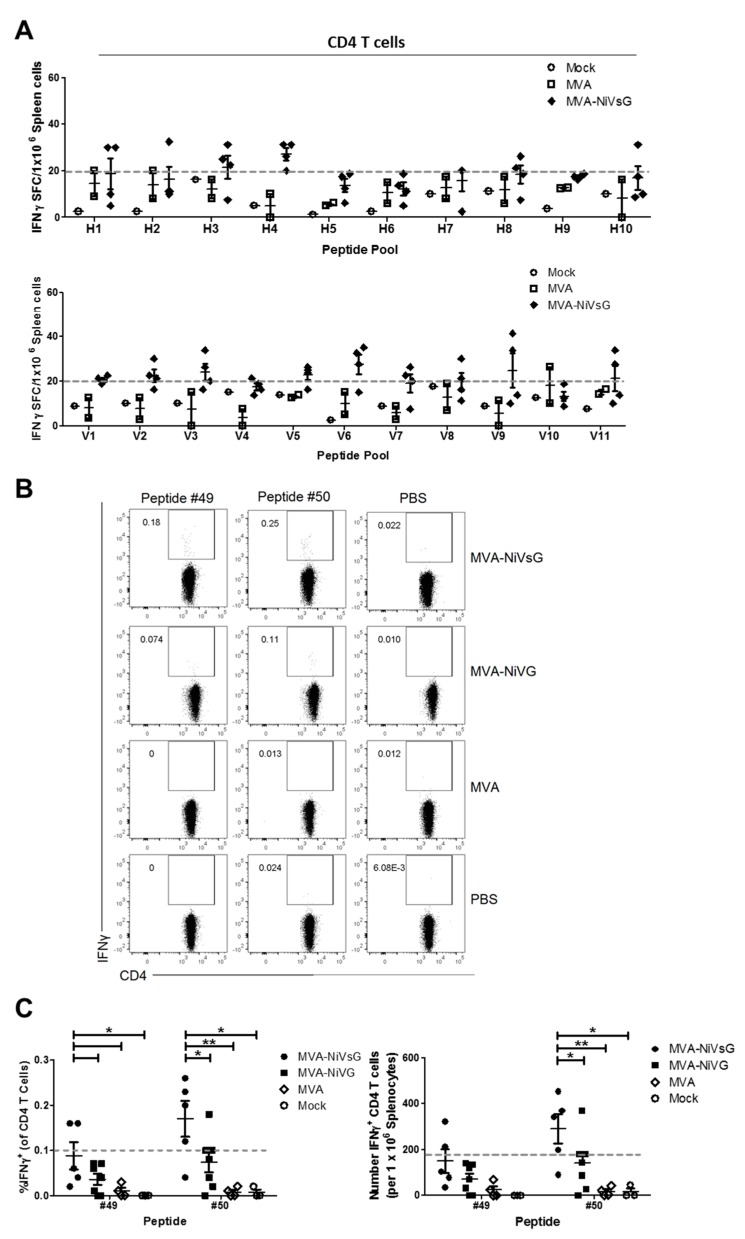
Activation of antigen-specific CD4 T cells after immunization with the recombinant MVA candidate vaccines expressing NiV-G or NiV-sG. Groups of IFNAR−/− mice (*n* = 2–7) were immunized twice with MVA–NiVsG, MVA–NiV-G, MVA, or saline (mock) via the i.p. or i.m. route over a 21 day period. Eight days after the final immunization, CD4 T cell-enriched splenocytes or total splenocytes were restimulated and measured by IFN-γ ELISPOT assay or IFN-γ intracellular cytokine staining (ICS) plus FACS analysis. (**A**) Screening for H2-IAb-restricted T cell epitopes of NiV-G in CD4 T cell-enriched splenocytes obtained from i.p. immunized mice. T cell responses were measured by IFN-γ ELISPOT assay. Graph shows IFN-γ SFC of CD4 T cell-enriched splenocytes stimulated with peptide pools H1–H10 and V1–V11. The grey dashed line represents the group mean cut off value (20 IFN-γ SFC/10^6^ cells) for identifying positive peptide pools. (**B,C**) Identification of H2-IAb-restricted candidate epitopes of NiV-G by IFN-γ ICS and FACS analysis. Total splenocytes from i.m. immunized mice were restimulated with two promising candidate H2-IAb-restricted 15mer peptides, #49 (LFMTNVWTPPNPNTV) and #50 (NVWTPPNPNTVYHCS), identified by peptide pool screening and in silico H2-IAb-binding predictions using the IEDB database. (**B**) Representative flow cytometry dot plots showing IFN-γ production in the splenic CD4 T cell compartment. (**C**) Frequency and absolute number (per 10^6^ splenocytes) of IFN-γ+ CD4 T cells. Dashed line on graphs represent the cut off for definitively positive samples. Differences between individual groups were analyzed by one-way ANOVA and Tukey post-hoc test. Asterisks represent statistically significant differences between two groups for a specific peptide. * *p* < 0.05, ** *p* < 0.01.

Initially, we also analyzed purified CD4 T cell cultures from MVA–NiVsG immunized mice for their ability to stimulate IFN-γ by ELISPOT using a two-dimensional pooled-peptide matrix system (Figure 3A, Section 3.4.1). In contrast to CD8 T cell-enriched splenocytes, the IFN-γ SFC signals in these cultures were lower (Figure 3B and Figure 6A). In order to determine definitively which peptide pools were above background levels, we selected a cut off value of 20 IFN-γ SFC/10^6^ cells (Figure 6A, grey line). Mean IFN-γ SFC values above background were observed in 8 out of the 21 pools (pools H3, H4, V2, V3, V5, V6, V9, and V11) (Figure 6A).

In order to identify potential CD4 T cell epitopes of H2-IAb, we performed a computational analysis of the 15mer peptides in the five most positive peptide pools measured by ELISPOT assay (pools H3, H4, V3, V6, and V9). Using MHC II binding predictions obtained from the IEDB online resource, we found two promising peptides. These peptides were #49 (LFMTNVWTPPNPNTV) and #50 (NVWTPPNPNTVYHCS) (Table 2). Next, we used IFN-γ ICS to identify directly antigen-specific CD4 T cells after stimulation with these peptides. For this, splenocytes from mice that had been vaccinated with MVA–NiV-G or MVA–NiVsG were stimulated with peptides #49 and #50 and analyzed by flow cytometry. A small population of peptide-specific IFN-γ+ CD4 T cells was observed in the MVA–NiVsG and MVA–NiV-G groups (Figure 6B). Due to low frequencies of IFN-γ-producing cells, we chose a cut off value of 0.1% to differentiate between positive signals and background. Overall, CD4 T cells from the MVA–NiVsG group had a frequency of IFN-γ+ above the cut off relative to MVA–NiV-G group (Figure 6C). The mean percentage of IFN-γ+ CD4 T cells for the two peptides was 0.09–0.17% and 0.04–0.07% for MVA–NiVsG and MVA–NiV-G, respectively. Moreover, the frequency and absolute number of IFN-γ-producing CD4 T cells in peptide #50 stimulated cultures was significantly higher in the MVA–NiVsG group when compared with the MVA–NiV-G group (mean = 290 ± 65 and 140 ± 46 cells/10^6^ splenocytes respectively). For peptide #49, MVA–NiVsG vaccinated mice showed a significantly elevated frequency of IFN-γ+ CD4 T cells only. In conclusion, our data indicate that peptide #50 (NVWTPPNPNTVYHCS) is a promising H2-IAb-restricted CD4 T cell epitope candidate of NiV-G. The alignment of the peptide to the full amino acid sequence of wild-type NiV-G is shown in Figure S3.

## 4. Discussion

The continuous threat of suddenly emerging NiV outbreaks, particularly in Bangladesh and India, demonstrate the need for countermeasure approaches ready to use in an immediate public health response. At present, there are no licensed NiV vaccines for use in humans available. The existence of a NiV candidate vaccine should significantly reduce the risk of infection and transmission of the virus in the case of an outbreak scenario. There are some experimental NiV vaccines that have already been tested in different preclinical animal models. The major focus of these approaches was to evaluate the immunogenicity and efficacy in the context of NiV challenge infection. In those studies, efficacy has been mostly associated with the generation of NiV-specific antibodies, and immune monitoring is mainly relying on the detection of virus-neutralizing antibodies [31,60,61]. However, there is relatively little known about the induction and the relevance of NiV-specific cellular immune responses. In that context, the availability of appropriate tools to investigate the role of T cells in NiV-specific immunity is an important prerequisite in the development of new vaccines and therapeutics. Thus, it will be indispensable to monitor in animal models the contribution of virus-specific T cells to protective immunity but also to potential NiV antigen-specific immune pathology.

Here, we identified a major histocompatibility complex (MHC) haplotype H2-b-restricted peptide epitope in the NiV-G protein by stimulating T cells from MVA−NiVsG vaccinated IFNAR−/− mice with a two-dimensional (2D) matrix pool of overlapping peptides. IFNAR−/− mice have been already established as a valuable preclinical animal model for NiV infection with a LD_50_ of 8 × 10^3^ pfu after intraperitoneal challenge infection [15]. Moreover, in previous studies, we have already successfully demonstrated that interferon type I receptor knockout mice (IFNAR−/−) [47] can be readily used as animal models to study the immunogenicity and protective capacity of MVA immunization [62,63]. Here, we wished to specifically assess the ability of MVA-delivered NiV-G antigen to induce the activation of cellular immune responses in mice. In general, the envelope G protein is known as the well-conserved attachment glycoprotein for both HeV and NiV. In a previous study, a recombinant adeno-associated virus vaccine expressing a full-length NiV-G protein protected hamsters in an NiV infection model [38]. In another approach, a soluble version of HeV-G has been engineered and showed an even more advantageous efficacy when tested in different preclinical animal models [42,43,64]. Using HeVsG, a monoclonal antibody m102.4 was derived and has already been successfully tested as a therapeutic approach in humans. To further enhance *Henipavirus* G protein-induced immunogenicity, we also designed and tested a soluble version of the NiV-G protein (NiVsG) similar to the HeVsG antigen used for the generation of m102.4. To comparatively evaluate the immunogenicity of the NiVsG protein, we also generated an MVA expressing full-length G. The recombinant viruses MVA–NiV-G and MVA–NiVsG produced stable amounts of NiV-G antigen upon in vitro infection of human cells, indicating the unimpaired expression of the target gene under transcriptional control of the synthetic vaccinia virus-specific early–late promoter PmH5 or the strong natural early promoter Pvgf. In the case of MVA–NiVsG, removal of the transmembrane domain and cytoplasmic tail resulted in the secretion of the NiV-G from MVA-infected cells and accumulation also in the supernatant of cell cultures, as demonstrated in Western blot analysis and immunostaining. A similar result was obtained with HeVsG, as expressed by conventional recombinant VACV [41]. In contrast, the full-length MVA-produced NiV-G protein was not released in the supernatant, indicating the stable presentation on the cell surface through the transmembrane domains. Another important aspect of proper protein expression, folding and conformational stability is influenced by the N-glycans. Recent studies indicated that NiV-G N-glycans reduce fusion efficiency because removal of some N-glycans caused cell–cell hyperfusogenicity and increased viral entry [65]. Glycosidase treatment of full-length MVA-produced NiV-G resulted in a polypeptide of 58 kDa, corresponding to the molecular mass predicted from the NiV G gene encoding sequences. The glycosidase treatment of the NiVsG also indicated the presence of all the N-glycans sites within the soluble version of the glycoprotein. A first in vivo evaluation in mice revealed that treatment with the MVA–NiV-G and MVA–NiVsG candidate vaccines resulted in the induction of similar levels of G-binding serum antibodies, confirming the immunogenicity of both antigen versions [66]. In that context, the presence of binding antibodies seems to play a substantial role in the blocking of NiV entry, since the mechanism of NiV neutralization is complex and involves more antigenic sites than those required for simple receptor binding [67]. However, more recent studies in different animal models suggest that cellular immune responses are also involved in mediating protection against NiV infection [38,68]. This observation is further supported by studies in a pig model for NiV infection showing substantial activation of CD8 T cells after oral infection with NiV [45]. In line with these preclinical data, humans surviving NiV infection [2] showed significant levels of proliferating (Ki-67+) CD8 T cells, indicating the presence of acute effector cells. These data emphasize that in addition to the humoral immune responses, T cells could be associated with recovery from NiV infection. In a more recent study, Stroh and coworkers confirmed the activation of NiV-specific CD8 T cells in mice after vaccination with NiV-like particles. These data further highlight that T cells may play a critical role in NiV infection [68]. Another hypothesis is that NiV-specific T cells could be involved in potential immunopathologies. In this context, data from infections with other neurotropic viruses, for example, West Nile virus, demonstrated that antigen-specific T cells can open the blood brain barrier and contribute to virus infections of the brain [69,70,71,72,73]. Thus, to allow for more detailed studies characterizing T cells in NiV-associated immunity or pathogenesis, it is essential to identify NiV-G peptide epitopes allowing for the specific MHC-restricted antigen presentation and the activation of NiV-specific T cells. We identified a nonamer epitope NiV-G-9.3_75–83_ (RSTDNQAVI). Analysis of this peptide sequence showed that NiV-G 9.3_75–83_ could be functionally conserved in Hendra virus, but not Cedar virus (another recognized *Henipavirus*), G antigens (Figure S3). Structural and functional analyses reveal promiscuous and species-specific use of ephrin receptors by Cedar virus [74]. This potential epitope will support a more detailed experimental characterization of T cells induced by NiV infection in the mouse model and their contribution for pathogenesis and protection. In this study, we found that MVA–NiVsG vaccination induced significantly higher amounts of NiV-G epitope-specific CD8 T cells compared with the MVA–NiV-G candidate vaccine. This was a somewhat surprising observation as the immunizations with both antigens had elicited very comparable levels of G-specific antibodies. It is tempting to speculate that NiVsG, as a soluble antigen, can trigger enhanced T cell responses because it is available to two different pathways of antigen presentation. On the one hand, NiVsG as intracellularly synthetized antigen is endogenously processed and presented via MHC-I on the cell surface to activate CD8 T cells. In addition, the NiVsG is secreted in high amounts from the MVA–NiVsG-infected cells, and such extracellular antigen can efficiently fuel the cross-presentation pathway and thereby induce elevated CD8 and CD4 T cell immune responses [75,76]. Interestingly, the MVA–NiVsG candidate vaccine also proved to rapidly induce NiV-G epitope-specific CD8 T cells after single-dose application. These data are of relevance, since the epidemiology of the more recent NiV outbreaks demonstrated that a potential vaccine candidate should rapidly protect. Importantly, an H2b-restricted epitope has been identified in IFNAR−/− mice, which serve as an established small animal model for NiV infection [15]. In addition, we showed the induction of NiV-G-specific CD4 T cells upon prime-boost immunization in the IFNAR−/− with the MVA–NiV vaccines and using peptides for in vitro stimulation, as identified by using MHC II binding predictions obtained from the IEDB online resource (www.iedb.org). This data goes well along with the hypothesis that CD4 T cell responses are also significantly elevated upon infection [77]. Again, MVA–NiVsG vaccination results in more efficient activation of CD4 T cell responses. Further experiments will be needed to characterize the contribution of NiV G-specific CD4 T cells for NiV infection in more detail. Taken together, our findings showed the activation of NiV-G-specific T cells in IFNAR−/− mice following vaccination with MVA-based candidate vaccines. We confirmed the identification of potential H2-b-restricted NiV-G CD8 and CD4 T cell peptide epitopes. In this study we also demonstrated that an MVA–NiVsG candidate vaccine may have superior immunogenicity, resulting in NiV-specific antibodies and T cells in IFNAR−/− mice. These data emphasize the promise of future studies in this animal model further evaluating the role of NiV-specific T cells activated by the G-9.3_75–83_ and G-50_269–283_ peptide epitopes, in both vaccine-induced protection and potential contribution to NiV-induced pathologies.

## Figures and Tables

**Table 1:** Information on 8–11mer peptides used for H2-b-restricted epitope screening.

Peptide ID	Sequence	Length	Start Position	End Position
#1	YT**RSTDNQAVI**KDAL	15	73	87
8.1	YTRSTDNQ	8	73	80
8.2	TRSTDNQA	8	74	81
8.3	RSTDNQAV	8	75	82
8.4	STDNQAVI	8	76	83
9.1	YTRSTDNQA	9	73	81
9.2	TRSTDNQAV	9	74	82
9.3	**RSTDNQAVI**	9	75	83
9.4	STDNQAVIK	9	76	84
10.1	YTRSTDNQAV	10	73	82
10.2	T**RSTDNQAVI**	10	74	83
10.3	**RSTDNQAVI** K	10	75	84
10.4	STDNQAVIKD	10	76	85
11.1	YT**RSTDNQAVI**	11	73	83
11.2	T**RSTDNQAVI**K	11	74	84
11.3	**RSTDNQAVI** KD	11	75	85
11.4	STDNQAVIKDA	11	76	86

Sequence of peptide 9.3 (RSTDNQAVI) highlight in bold and sequence of peptide 8.4 (STDNQAVI) shown by the underline. Sequences of positive 8-11mer peptides are indicated in blue.

**Table 2:** Information on 8–11mer peptides used for H2-b-restricted epitope screening.

Peptide Name	Peptide ID	Sequence	Length	Start Position	End Positions	MHC Restriction
G-1	#1	YTRSTDNQAVIKDAL	15	73	87	H2-b
**G-9.3**	**9.3 ^1^**	**RSTDNQAVI**	**9**	**75**	**83**	**H2-Db**
G-49	#49	LFMTNVWTPPNPNTV	15	265	279	H2-IAb
**G-50**	**#50 ^2^**	**NVWTPPNPNTVYHCS**	**15**	**269**	**283**	**H2-IAb**

^1^ Peptide chosen as the most promising H2-b-restricted peptide of NiV-G; ^2^ Peptide chosen as the most promising H2-IAb-restricted peptide of NiV-G. Rows in bold indicate promising NiV-G-specific T cell epitopes.

## Supplementary Materials

The following are available online at https://www.mdpi.com/1999-4915/12/1/26/s1, Figure S1: Generation and characterization of recombinant MVA–NiVsG and MVA–NiV-G, Figure S2: IFN-γ production by CD8 and CD4 T cells stimulated by the NiV-G peptide 9.3, Figure S3: Amino acid alignment of H2-b- and H2-IAb-restricted peptides, Figure S4: Quality of activated CD8 T cells from mice immunized with recombinant MVA expressing NiV-G.
