# Supplementary figures and images for: A Soluble Version of Nipah Virus Glycoprotein G Delivered by Vaccinia Virus MVA Activates Specific CD8 and CD4 T Cells in Mice
Source: Viruses. 2019 Dec 24;12(1):26. doi: 10.3390/v12010026 (PMC7019319; doi:10.3390/v12010026)

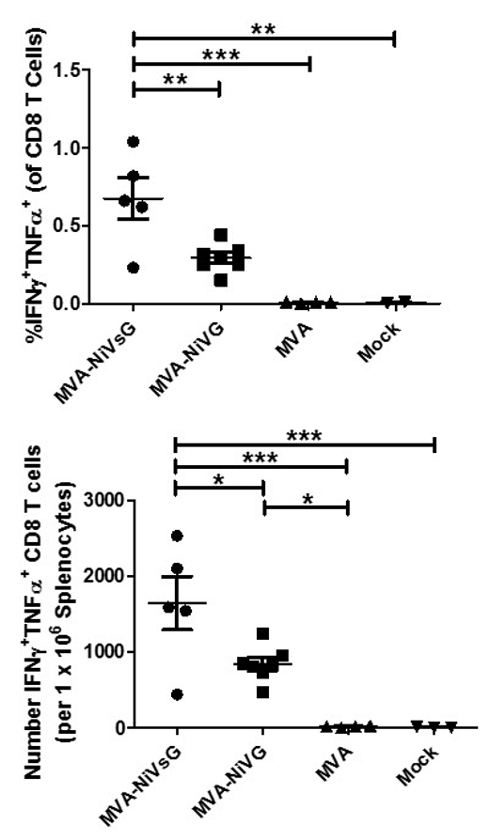

Supplement: Supplementary file 1 [file viruses-12-00026-s001.zip › Figure S4B.jpg]

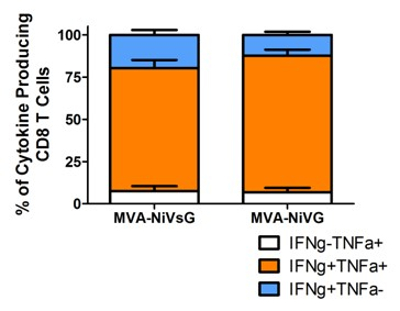

Supplement: Supplementary file 1 [file viruses-12-00026-s001.zip › Figure S4C.jpg]

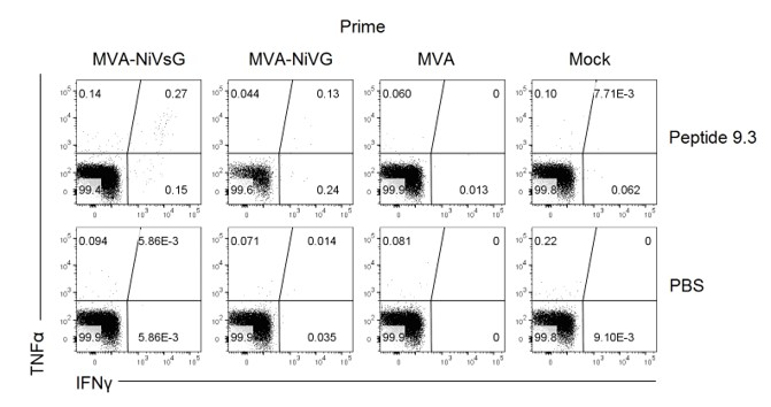

Supplement: Supplementary file 1 [file viruses-12-00026-s001.zip › Figure S4D.jpg]

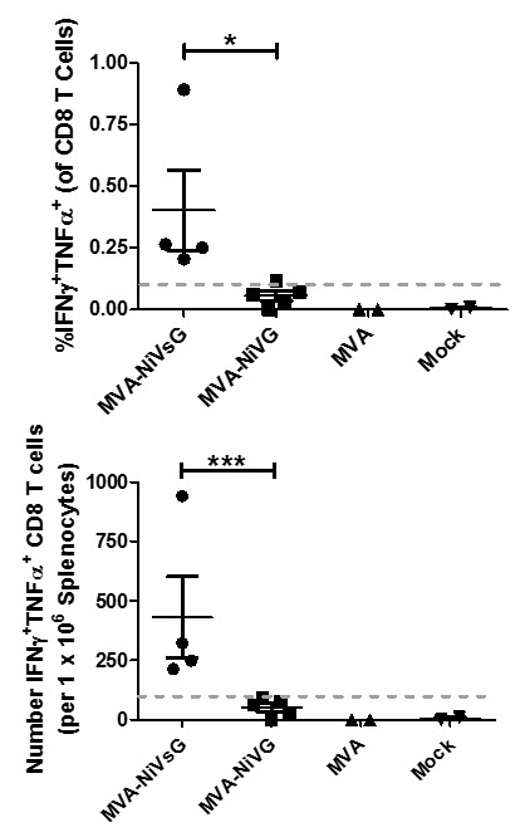

Supplement: Supplementary file 1 [file viruses-12-00026-s001.zip › Figure S4E.jpg]

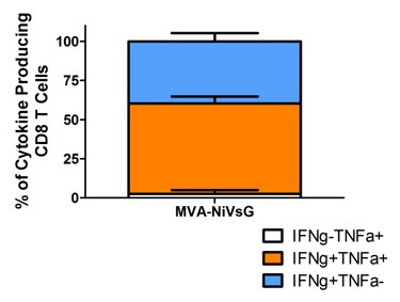

Supplement: Supplementary file 1 [file viruses-12-00026-s001.zip › Figure S4F.jpg]

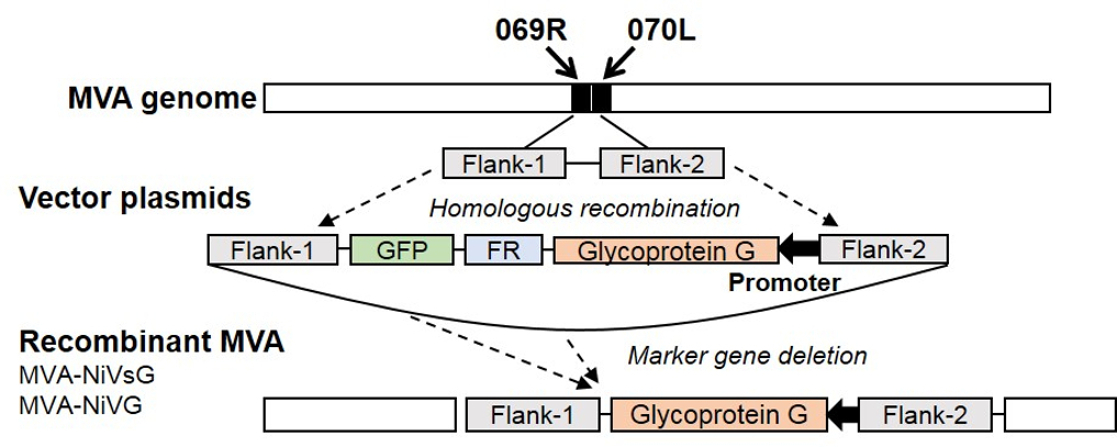

Supplement: Supplementary file 1 [file viruses-12-00026-s001.zip › Figure S1A.jpg]

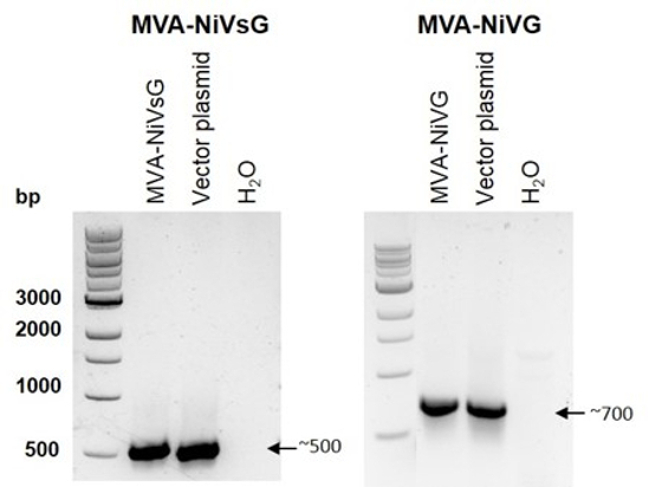

Supplement: Supplementary file 1 [file viruses-12-00026-s001.zip › Figure S1B.jpg]

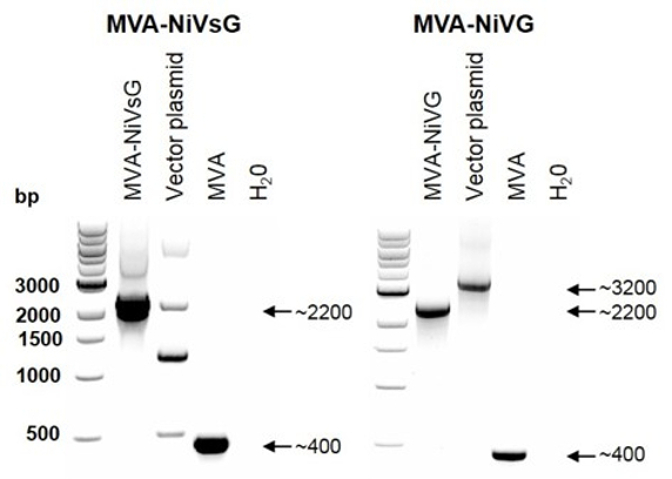

Supplement: Supplementary file 1 [file viruses-12-00026-s001.zip › Figure S1C.jpg]

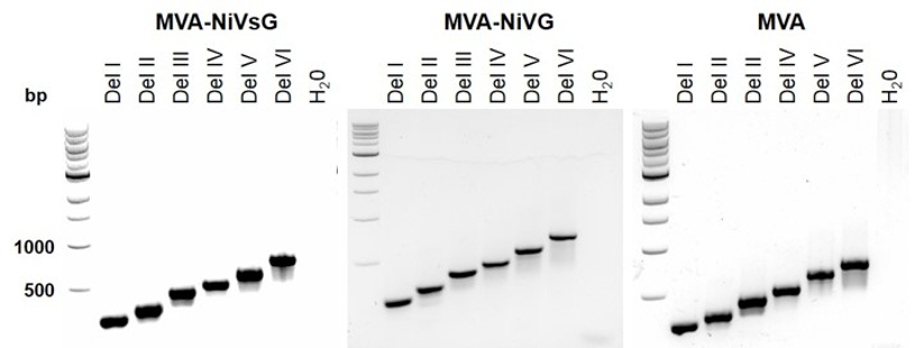

Supplement: Supplementary file 1 [file viruses-12-00026-s001.zip › Figure S1D.jpg]

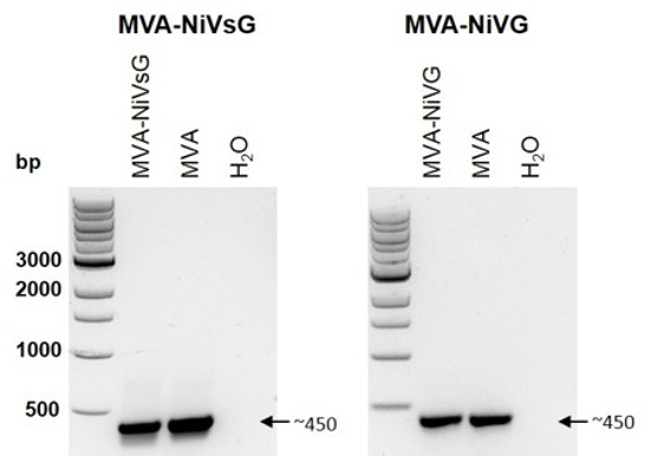

Supplement: Supplementary file 1 [file viruses-12-00026-s001.zip › Figure S1E.jpg]

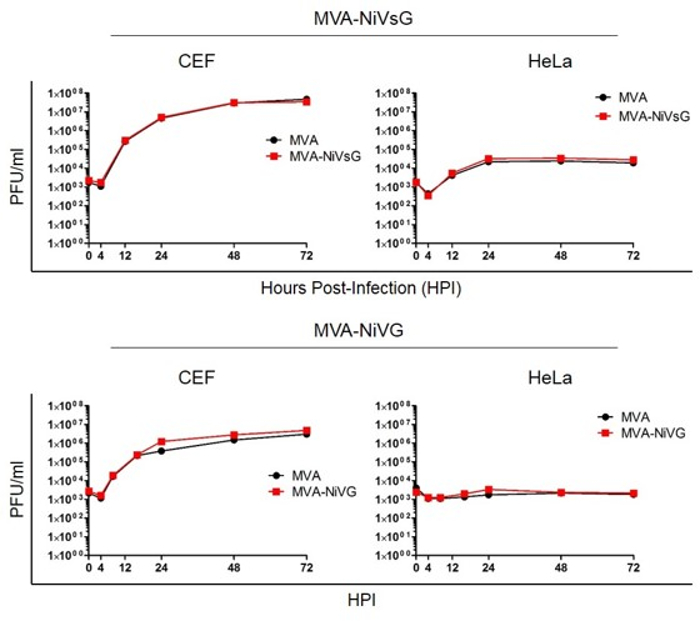

Supplement: Supplementary file 1 [file viruses-12-00026-s001.zip › Figure S1F.jpg]

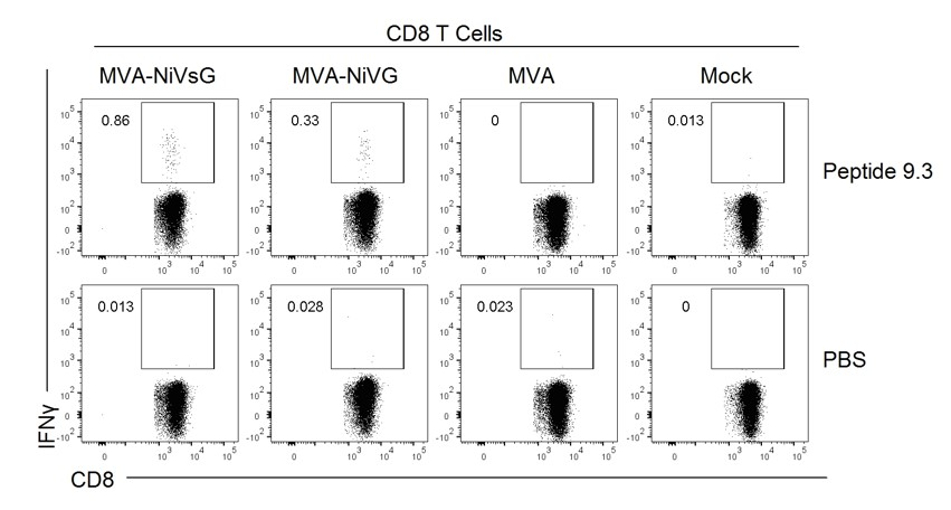

Supplement: Supplementary file 1 [file viruses-12-00026-s001.zip › Figure S2A.jpg]

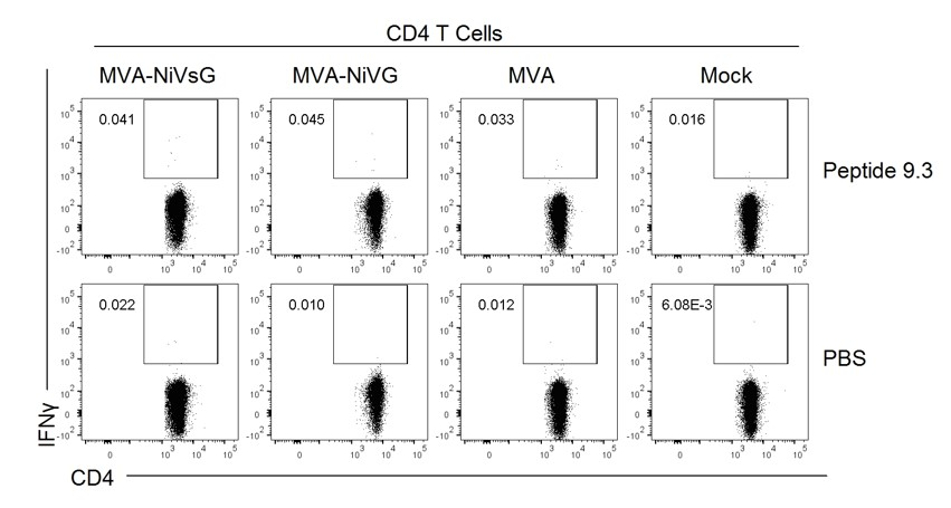

Supplement: Supplementary file 1 [file viruses-12-00026-s001.zip › Figure S2B.jpg]

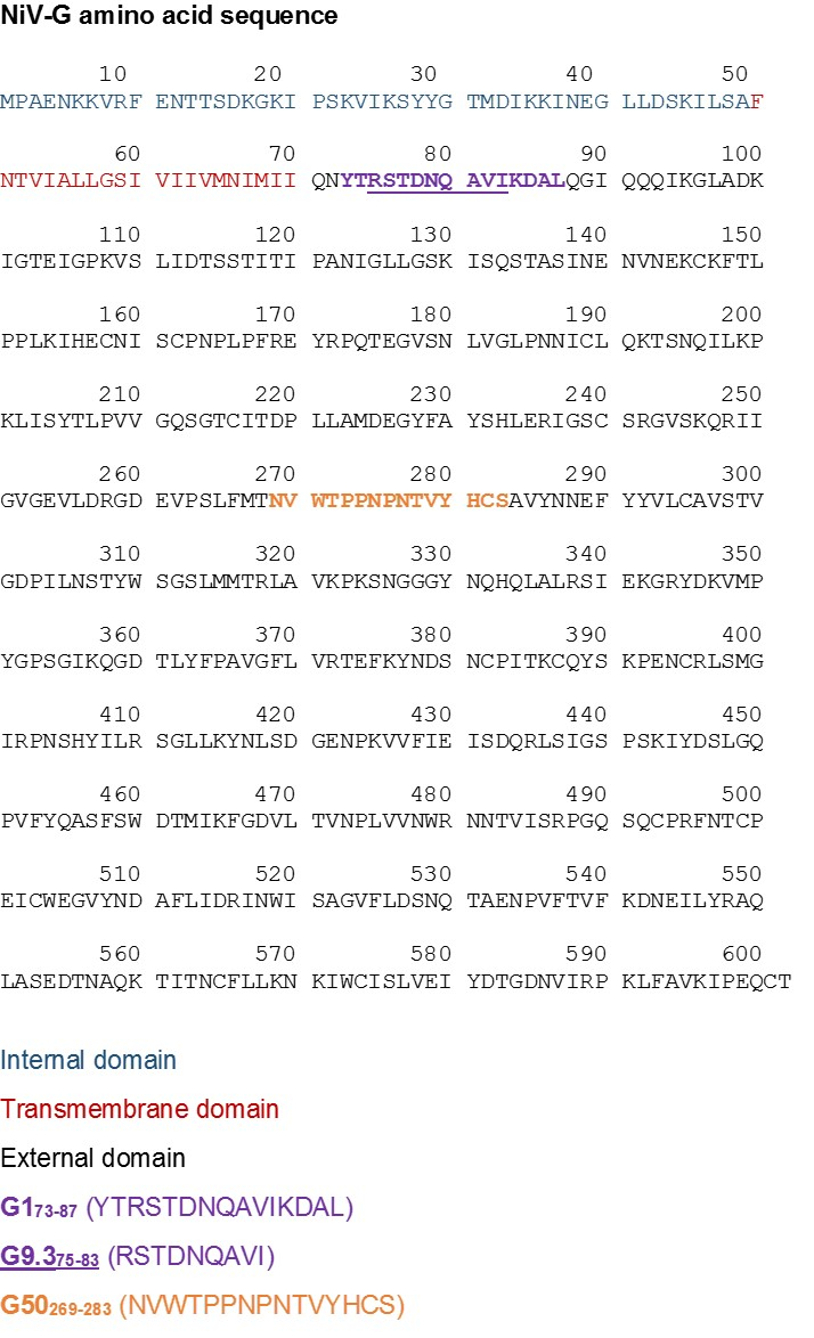

Supplement: Supplementary file 1 [file viruses-12-00026-s001.zip › Figure S3A.jpg]

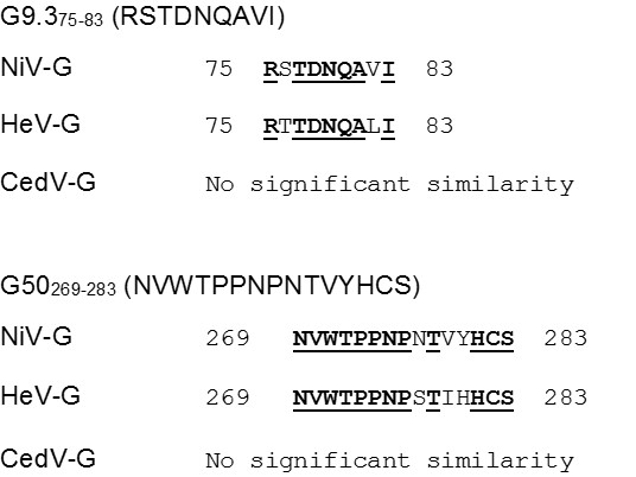

Supplement: Supplementary file 1 [file viruses-12-00026-s001.zip › Figure S3B.jpg]

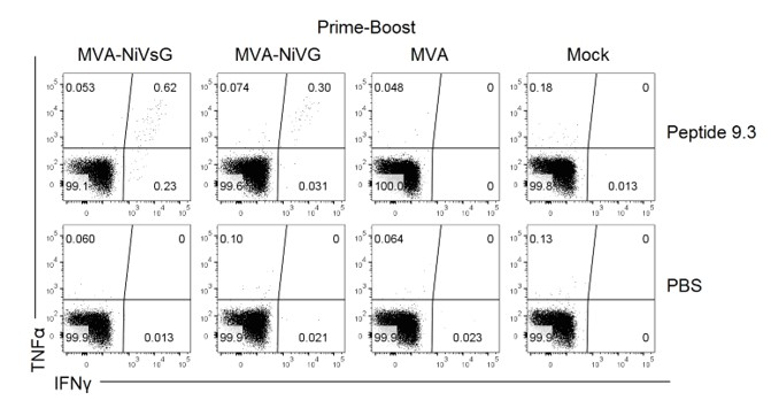

Supplement: Supplementary file 1 [file viruses-12-00026-s001.zip › Figure S4A.jpg]
